# A break from the pups: The effects of loft access on the welfare of lactating laboratory rats

**DOI:** 10.1371/journal.pone.0253020

**Published:** 2021-06-08

**Authors:** Anna S. Ratuski, Daniel M. Weary

**Affiliations:** Faculty of Land and Food Systems, Animal Welfare Program, University of British Columbia, Vancouver, BC, Canada; Istituto Superiore Di Sanita, ITALY

## Abstract

Rats (*Rattus norvegicus*) bred for research are typically confined with their litters until weaning, but will spend time away from pups when given the opportunity. We aimed to assess how dam welfare is affected by the ability to escape from their pups. Rat dams (n = 16) were housed in cages either with or without an elevated loft. We measured time dams spent in lofts, time spent nursing, and affective states using elevated plus maze and anticipatory behavior testing. We predicted that 1) dams housed with lofts would use them increasingly as pups aged, 2) dams without a loft would spend more time passively nursing (i.e. initiated by pups rather than the dam) and more total time nursing as pups aged, and 3) dams housed with lofts would show evidence of a more positive affective state. Dams housed with lofts spent more time in the loft with increasing pup age; dams spent on average (mean ± SE) 27 ± 5% of their time in the loft when pups were 1 wk old, increasing to 52 ± 5% of their time at 3 wks. When pups were 3 wks old, dams with lofts spent less time passively nursing (10 ± 2% of total time, compared to 27 ± 4% for dams without a loft) and less time nursing overall (36 ± 4% of time versus 59 ± 2% for dams without a loft). Rats without loft access showed increased anticipatory behavior potentially indicative of negative affective state (24.5±1.8 behaviors per minute in wk 3 compared to 18.8±1.0 in wk 1). These findings indicate that rat dams in laboratories choose to spend time away from their pups when provided the opportunity, particularly later in lactation; an inability to do so is associated with increased passive nursing and negative affect.

## Introduction

Laboratory rats (*Rattus norvegicus*) bred for research are typically confined with their litters until weaning. Under more natural conditions, rats raise their pups in burrow systems and can come and go when they choose, allowing the dam to gradually reduce the maternal care she provides. When given the choice, dams in a laboratory setting spend about 85% of their time with pups the day following parturition, but this decreases to about 30% by day 21 [1]. Standard laboratory cages limit the dam’s ability to avoid contact with her pups, perhaps affecting her welfare. Agency (i.e. control over one’s environment or the ability to escape stressors) is important for well-being, allowing for increased species-typical behaviors and helping animals avoid unpleasant conditions [2,3]. The lack of space and structural complexity in standard cages limits the dam’s ability to exert control over her environment, including escape from her pups.

Previous work on a variety of species has suggested that allowing dams to escape their litters is beneficial. For example, sows housed with access to a get-away area nursed less frequently and lost less weight than sows confined with piglets [4]. When given the opportunity to spend time away, sows spent less time with piglets later in lactation [4,5]. Permitting sows to reduce udder stimulation, and thus milk transfer before weaning, also helped to reduce stress and weight loss in piglets at weaning [6]. In another example, mink dams with get-away bunks spent more time away as kits grew older, showed reduced stereotypic behaviors, and had improved teat conditions [7]. Finally, mouse dams housed in standard laboratory cages with an elevated get-away tunnel showed lower pup mortality than standard-housed mice [8]; pup mortality can also be reduced using other enrichment strategies (e.g. [9]).

There is evidence that get-away housing could also benefit rat dams. Previous studies suggest that rat dams prefer to spend time away from the litter as pups grow older [1,10,11]. Cramer et al. [12] found that dams housed with pups showed high levels of press posture (i.e. pressing ventral surface against the floor or walls of the cage) when pups reached 14 days of age, averaging two bouts of press posture per day between days 14 and 32. Press posture behavior is also more likely when dams and their litters are housed in smaller cages [13], suggesting that dams attempt to limit nursing when unable to escape their pups. Prior studies allowing for rat dams to spend time away from pups have focused on patterns of maternal behavior and time spent with the litter (e.g. [1,10,11,14]). Two nursing styles have been identified: active (which involves the dam hovering over pups and tends to occur with licking/grooming by the dam), and passive (where the dam is typically recumbent) [15]; prior studies have not yet assessed how the ability to escape pups affects the nature of nursing interactions. The inability to perform motivated natural behaviors such as avoiding nursing attempts of older pups, and more generally the lack of ability to control their environment (i.e. lack of agency) may have detrimental effects on the affective states and overall welfare of animals [16].

Affective states in rats have been assessed using a variety of techniques (see [17] for a review). For the current study we used the elevated plus maze (EPM) and anticipatory behavior testing. The EPM is commonly used to assess anxiety in rodents; animals that spend more time in open arms of the maze are considered less anxious [18]. Anticipatory behavior is often characterized by an increased frequency of behavioral transitions (i.e. switching from one behavior to another) following the presentation of a conditioned stimulus as the rat awaits the provision of a reward [19,20]. Anticipatory testing can be used to make inferences about an animal’s reward sensitivity, with the prediction that rats living in poor environmental conditions show increased sensitivity to rewards and thus will express more anticipatory behavior [20,21].

The aim of this study was to assess if providing dams with the opportunity to spend time away from pups would lead to improved affective states and altered maternal interactions. Rats were housed in commercially available cages with removable elevated lofts and allocated to either a loft or no loft treatment to manipulate their ability to spend time away from pups. We predicted that 1) dams with access to a loft would use it increasingly as pups aged, 2) dams housed without a loft would spend more time passively nursing as pups aged, and 3) dams would show evidence of a more positive affective state when housed in the loft treatment, especially as pups aged, as evidenced by spending more time in the open arms of the EPM and by showing fewer anticipatory behaviors.

## Materials and methods

All procedures were approved by the Animal Care Committee (protocol A18-0034) at The University of British Columbia.

### Subjects and experimental design

We used 18 primiparous female Sprague-Dawley rats obtained as surplus stock from within the university. Animals were not time mated, so allocation to treatments was staggered rather than occurring all at once, with rats randomly assigned to treatments while pregnant. Two rats from the no loft treatment had to be euthanized during the study due to illness, resulting in a final sample size of 16 rats (n = 9 in the loft treatment and n = 7 in the no loft treatment). Rats were aged 9 months (n = 6; evenly distributed between treatments) and 4 months (n = 10; 6 in the loft group and 4 in the no loft group) at the time of testing. Before training began for any test, we habituated all of the rats to the presence of researchers over approximately 2–3 weeks by passively placing one hand in the cage and by feeding treats (Froot Loops cereal; Kellogg’s, Mississauga, Canada) several times per week.

**Fig 1:**
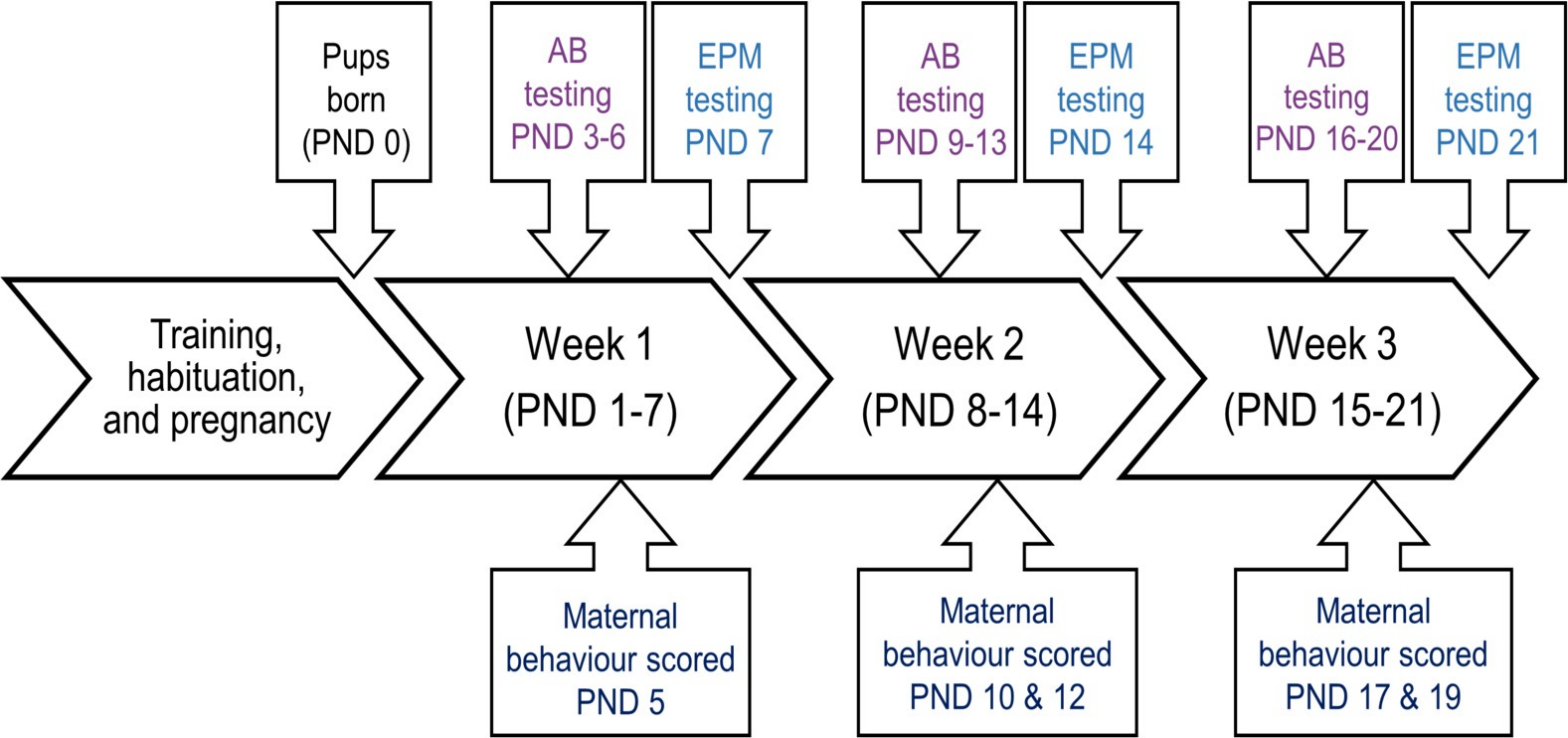
Experimental timeline for anticipatory behavior (AB), elevated plus maze (EPM) and maternal behavior data collection. “Training” refers to anticipatory behavior conditioning, while “habituation” refers to initial exposures to the experimenters, the elevated plus maze, and removal of lofts for rats in the no loft treatment. Time is shown relative to the day pups were born (Post-natal day, PND, 0).

Our experimental treatment could be framed as loss of the loft rather than access to a loft, given that all rats were housed with lofts initially and some had them removed for the experiment. Rats in the no loft treatment had lofts removed from their cages approximately 5 days before they were expected to give birth (i.e. post-natal day, PND, 0), while rats in the loft treatment kept the loft in their cage. Litter sizes were limited to 12 pups (if a dam gave birth to more than 12 pups, extra pups were euthanized by PND 3), as this was the average litter size in a previous study [13]. Six litters consisted of fewer than 12 pups (4 in the loft treatment and 2 in the no loft treatment). In one case when extra pups of the same age were available, extra pups from a larger litter were cross-fostered on PND 3 to increase the size of a smaller litter (S1 Table). Data collection began on PND 3 and ended on PND 21; Fig 1 provides a timeline of the experiment. At the end of the study, 8 of the dams were donated for use in a teaching protocol at the University of British Columbia. All remaining dams were euthanized in their home cages using isoflurane anesthesia followed by carbon dioxide.

### Housing

Rats were housed in cages with or without removable lofts that provide an upper level in the cage (Optirat Plus, Animal Care Systems, Colorado, USA; 38.9 cm x 56.9 cm x 26.2 cm). This resulted in a cage floor area of 1181 cm^2^ for cages without the loft and 1574 cm^2^ for cages with the loft (S1 Fig). Water was available *ad libitum*, as was food (Teklad Rat Diet 2918, Envigo, IN, USA), placed on the cage floor. All cages contained aspen chip bedding (Northeastern Products Corp., NY, USA) and a combination of paper towels and crinkle paper as nesting material (Enviro-Dri, Shepherd Specialty Papers, TN, USA). Rats were pair-housed in these cages for the entirety of their lives with the exception of the breeding period. For breeding, females were randomly paired with males and housed in male-female pairs for 7–10 days in standard rectangular cages (Bioscape; 43 cm x 30 cm x 18 cm) and then singly housed in the Optirat Plus cages while pregnant. Rats were housed on a 12-h light cycle (light from 7:00–19:00 h) with a mean room temperature of 22.3 ± 0.08 ⁰C and a mean humidity of 40.1% ± 5.89. Rats were weighed on PND 7, 14, and 21 (S2 Table).

### Maternal behavior

Home cage behavior was video-recorded using a DVR (Amcrest ProHD 8CH Digital Recorder). The ethogram for maternal behavior followed Macrì et al. [22] and Myers et al. [23]. Videos were scored for target behaviors (outlined in Table 1) by two observers blind to rat identity and research predictions. Behavior was scored on PND 5, 10, 12, 17, and 19, selected based on the expected trends in maternal care (e.g. [1]), and to avoid days when EPM testing was conducted.

**Table 1:** Descriptions of dam behaviors scored in home cage videos.

Behavior	Definition
**Active nursing**	Dam is standing over pups and nursing with arched-back posture (i.e. dam’s back appears rounded and her weight is supported by her legs) and legs spread apart, or dam is nursing in seated or blanket posture (i.e. laying over pups with back flat rather than arched) while grooming pups
**Passive nursing**	Also known as supine nursing; dam is sitting or laying on her back or side while nursing pups. Alternatively, pups are latching onto dam and trying to nurse while she is otherwise occupied (e.g. walking, eating, drinking, or grooming herself)
**In loft**	Dam is in the loft (all four feet)

Rat dams show a daily rhythm in nursing behavior, with higher nursing frequencies during the light period and reduced contact during the dark period [11,14]. We were interested in changes in maternal behavior and time spent away from pups, so behavior was scored in both the light and dark period. Videos were scored continuously for 1 h every 4 h, for a total of 6 h/d. The same hours were scored for all cages, with 3 h in the dark period and 3 h in the light (hrs 00, 04, 08, 12, 16, and 20). In the event that a video recording was unusable (e.g. due to technical difficulties with the DVR or a person entering the room and manipulating the cage rack), the hour immediately before or after was used whenever possible, or the hour was excluded. Of 480 videos, 64 were excluded. Interobserver reliability was assessed from a subset of randomly selected rats in each treatment for each of the days observed (active nursing r = 0.89, passive nursing r = 0.94, time spent in loft r = 0.99).

### Anticipatory behavior

Anticipatory behavior training began approximately 5 weeks before mating. Training occurred once per day, 5 days per week. Once rats had reached the end of the training schedule, they were trained at least twice per week leading up to parturition to maintain their conditioned response to the cue. The cage was placed on a cart for 1 min before the plastic lid to the home cage was removed and replaced with a wire lid resting on top of the cage. The wire lid made lofts inaccessible during anticipatory behavior testing, so rats from each treatment had the same amount of available area for movement. The experimenter then dragged a finger across the wire lid and started a timer, which made an audible beep. Rats were trained to associate this cue (finger rattle followed by beep) with the provision of a sweet food reward (two Froot Loops). During the first 10 sessions, the reward was given to the rat immediately following the cue. The time between the cue and the provision of the reward gradually increased to 3 min over the following 15 trials (S3 Table).

Anticipatory behavior was tested during the light phase for each rat twice on PND 3–6, PND 9–13, and PND 16–20, resulting in 6 trials/rat. Individual trials were conducted on separate days. Behavior was video recorded (Panasonic HD camcorder) and scored using BORIS observational software (Version 7.0.9; [24]). Observers were blinded to rat identity and treatment. Frequency and duration of behaviors were scored using an anticipatory behavior ethogram adapted from van der Harst and Spruijt [20] and Makowska and Weary [21] (Table 2). Interobserver reliability was assessed from 18 randomly selected trials scored by a second observer (total frequency of behavioral transitions r = 0.93).

**Table 2:** Ethogram used to score anticipatory behavior.

Behavior	Description
**Yawn**	Rat briefly opens mouth wide and inhales
**Walk**	Locomotion in any direction; all four paws are moving (no sniffing)
**Walk-sniff**	Locomotion in any direction while sniffing; all four paws are moving and rat is lifting nose in the air, minor up-down head movements
**Stretch**	Rat elongates limbs and abdomen; arches back
**Sit**	Resting on hind quarters with all four paws on the ground; rat may be looking around, sniffing, or pivoting without moving hind quarters
**Rear**	Rat stands up on hind legs or vertically stretches
**Rear-move**	While in rearing position, rat moves both front paws into new position
**Lie Down**	Rat’s abdomen is resting on flat surface; body not supported by paws
**Groom (self)**	Maintenance behaviors; face washing, coat cleaning, scratching self
**Eat**	Rat is holding a food pellet or sitting and eating
**Dig**	Rapid, successive movements of front and/or back paws while displacing bedding
**Alert**	Rat’s head raised suddenly; body and head still; body appears tense
**Hidden (out of sight)**	Rat is out of view; precluding observation
**Nesting material manipulation**	Rat is interacting with nesting material; carrying or pushing material around with head and/or forearms
**Pup interaction**	Dam is carrying pups or providing active maternal care

“Rear-move” was only scored when rats were already scored as rearing. Any instances of the behavior “hidden” were excluded from the total behavioral frequencies and trial duration.

### Elevated plus maze

The elevated plus maze (EPM) was 40 cm above the ground with four arms in the shape of a plus sign; each arm was 10 cm wide and 50 cm long. The two closed arms had walls 60 cm high, and the two open arms had no walls. All arms and walls were made of black acrylic glass, supported by a plastic base. Rats were habituated to the test in three separate trials before parturition, to account for changes in locomotion related to the initial novelty of the test, as behavior in the EPM tends to change between the first and subsequent exposures [25,26]. Dams were then tested in the EPM during the light phase on PND 7, PND 14, and PND 21. Trials lasted 5 min and were video recorded (Panasonic HD camcorder). The apparatus was cleaned with 70% isopropyl alcohol between trials. Dams were transported to the apparatus using agency-based handling, meaning dams willingly entered a transport box which was then moved to the centre of the apparatus, where they would then voluntarily exit the transport box to begin the test.

Videos were scored for time spent on outer or inner arms of the EPM by an observer blind to treatment and rat identity. This involved scoring the location of the front half of the rat’s body (i.e. wherever her head and both front legs were located) on either the open or closed arms of the EPM; a rat seated in the centre square of the EPM was scored as being on the closed arms unless the front half of the rat’s body extended onto one of the open arms. Interobserver reliability was assessed from 10 randomly selected trials scored by a second observer (r = 0.98 for time spent in open arms).

### Statistical analysis

Data analysis was conducted using SAS software (Version 9.4, SAS Institute Inc.) and plots were generated using the ggplot2 package in RStudio (R version 4.0.1). For all experimental outcomes, distribution of residuals were scrutinized and no transformations were deemed necessary. In preliminary analyses we visually assessed results according to dam age and found no clear differences between dams 4 and 9 months old. We also compared litters with 12 pups (n = 10) versus litters of <12 pups (n = 6) in preliminary analysis and again no differences were identified for any result. Our experiment was not powered to detect interactions between treatment and litter size or dam age. For each experimental outcome, the effect of treatment, pup age, and the interaction between treatment and pup age were included as fixed effects in mixed models with an autoregressive covariance structure, including rat identity as a random intercept.

To account for variable numbers of hours scored for each rat due to some missing videos, nursing behaviors were analyzed as proportions of total observation time. Week 1 values were based on observations on PND 5 only, while Weeks 2 and 3 represent the means of PND 10 and 12, and PND 17 and 19, respectively. All videos were missing for one rat on PND 12 and for another rat on PND 17 and 19, otherwise each rat had at least 4 hours scored for each day. Two outliers were identified for nursing behavior outcomes; these values were double checked and retained in analysis. Loft use was analyzed as a function of pup age for rats in the loft treatment only. The relationship between mean weekly nursing time and mean loft use was evaluated using Pearson correlation.

Anticipatory behavior results were analyzed as frequency of behavioral transitions per minute. Behavioural transitions were averaged per week for each rat. Data for the EPM were analyzed as proportion of time rats spent in the open arms per trial. Two observations were missing resulting in a total of 46 observations in the analysis of the EPM.

## Results

### Loft use and nursing behavior

**Fig 2:**
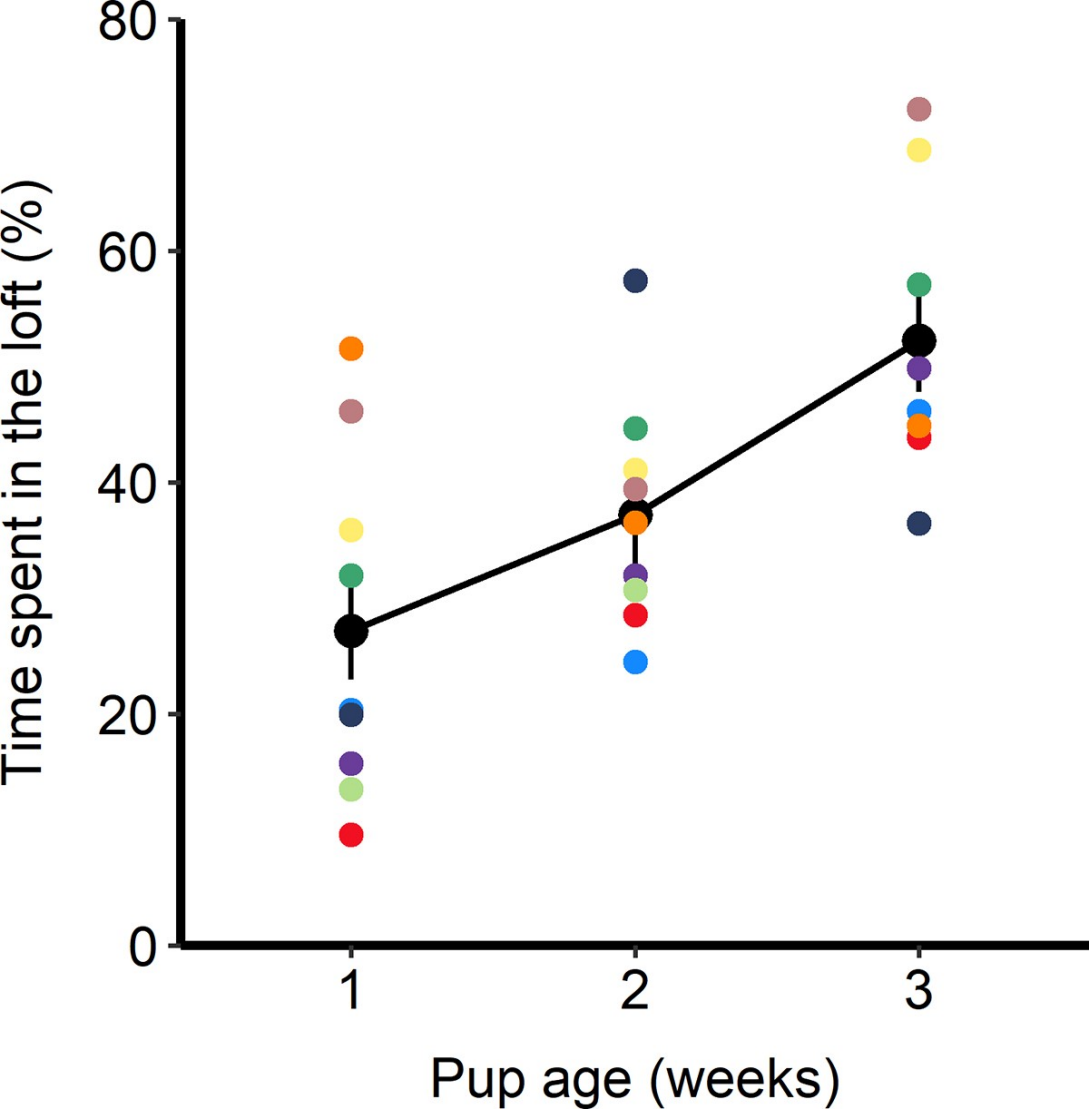
Mean time spent in the loft per week, expressed as a percentage of observational scans, for rats with loft access. The large black points and vertical bars depict least-square (LS) means and standard errors from the model output; smaller colorful points show the means of individual rats (n = 9 rats).

Loft use varied according to pup age (F_1,16_ = 17.43, p<0.01). Rats in the loft treatment made use of the lofts increasingly as pups became more mobile; mean time spent in the loft roughly doubled from week 1 to 3, from 27 ± 5% to 52 ± 5% of time spent in the loft (Fig 2).

**Fig 3:**
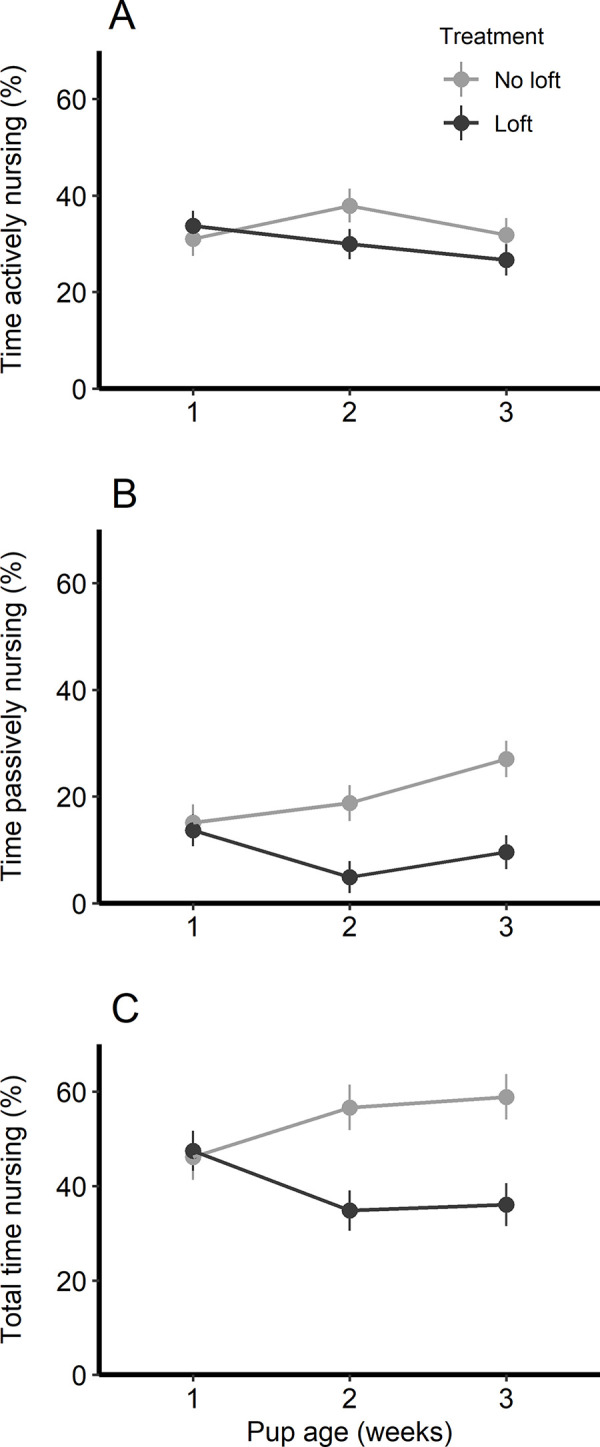
Mean time rats spent nursing per week, shown separately for A) active nursing, B) passive nursing, and C) total nursing. Results are shown separately by treatment, with 9 rats in the loft treatment and 7 rats in the no loft treatment. Plot depicts the LS means and standard errors from the model output.

**Fig 4:**
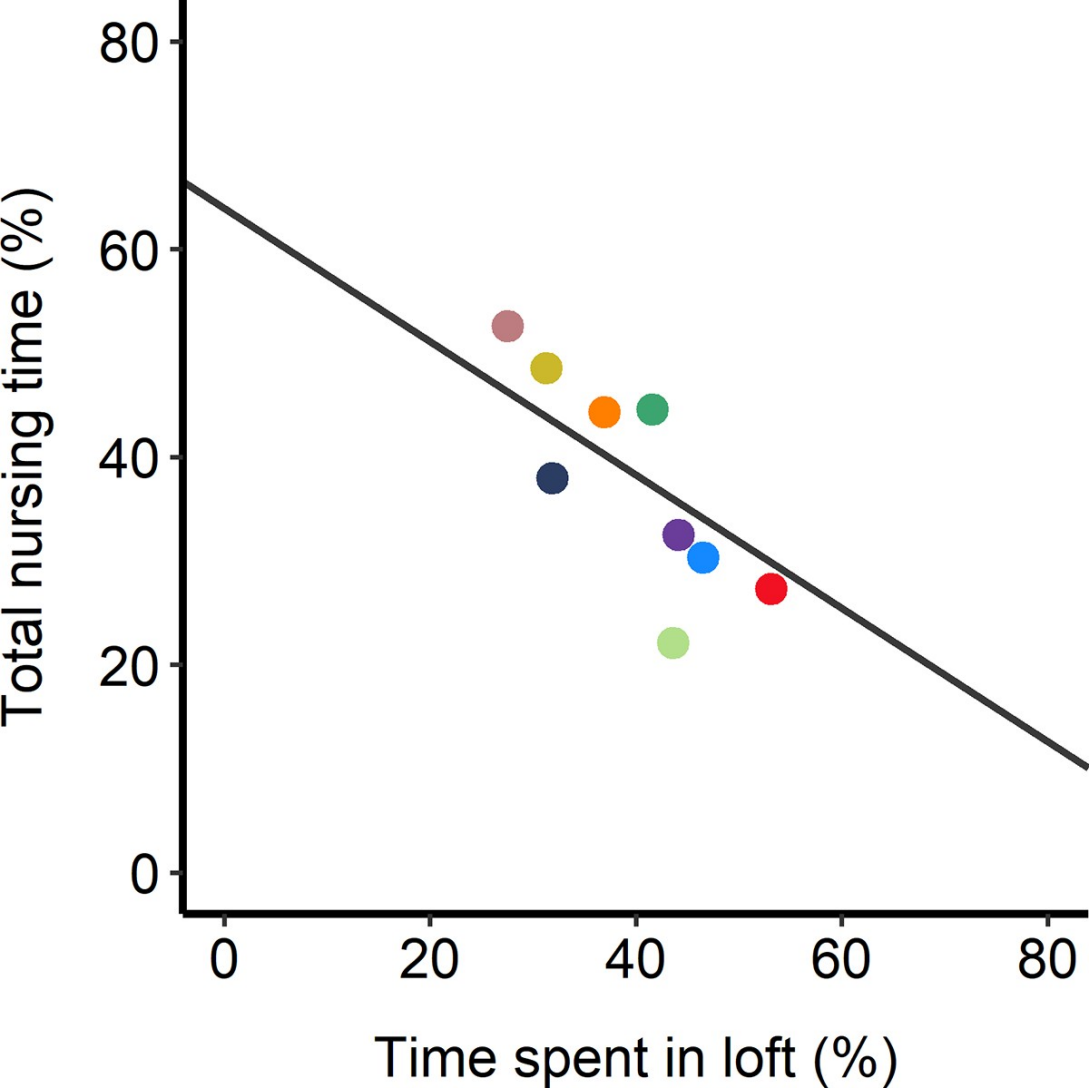
Correlation between total nursing time and loft use. Only rats from the loft treatment are shown. Each point represents the mean value for each rat (n = 9); individual rats are shown using different colours.

Time spent actively nursing did not vary with loft access (F_1,14_ = 0.78, p = 0.39), pup age (F_1,29_ = 1.2, p = 0.28), or the interaction between treatment and pup age (F_1,29_ = 1.5, p = 0.23; Fig 3). Rats in the loft treatment spent an average of 34 ± 3% of their time actively nursing in wk 1, and 27 ± 3% in wk 3; rats without lofts spent 34 ± 4% and 32 ±4%, respectively. In contrast, access to the loft decreased passive nursing and total nursing time, especially after week 1, driving a significant interaction for both measures (F_1,29_ = 6.12, p = 0.02, and F_1,29_ = 7.11, p = 0.01, respectively). When analyzed separately by treatment, pup age was associated with increased time spent passively nursing for dams without access to a loft (F_1,13_ = 5.84, p = 0.03), but not for rats with loft access (F_1,16_ = 0.99, p = 0.33). When pups were 3 wks old, dams with lofts spent less time passively nursing (10 ± 2% of total time, compared to 27 ± 4% for dams without a loft). Similarly, total time spent nursing increased with pup age for dam without lofts (F_1,13_ = 3.97, p = 0.07), reaching 59 ± 2% in wk 3. Total nursing time tended to decline with pup age for dams with access to a loft (F_1,16_ = 3.36, p = 0.09), reaching on average 36 ± 4% of time spent nursing at wk 3. Mean time spent in the loft was negatively correlated with mean time spent nursing (r = -0.80, p<0.01, n = 9; Fig 4), suggesting that loft use allowed the dams to better control nursing interactions.

### Anticipatory behavior

**Fig 5:**
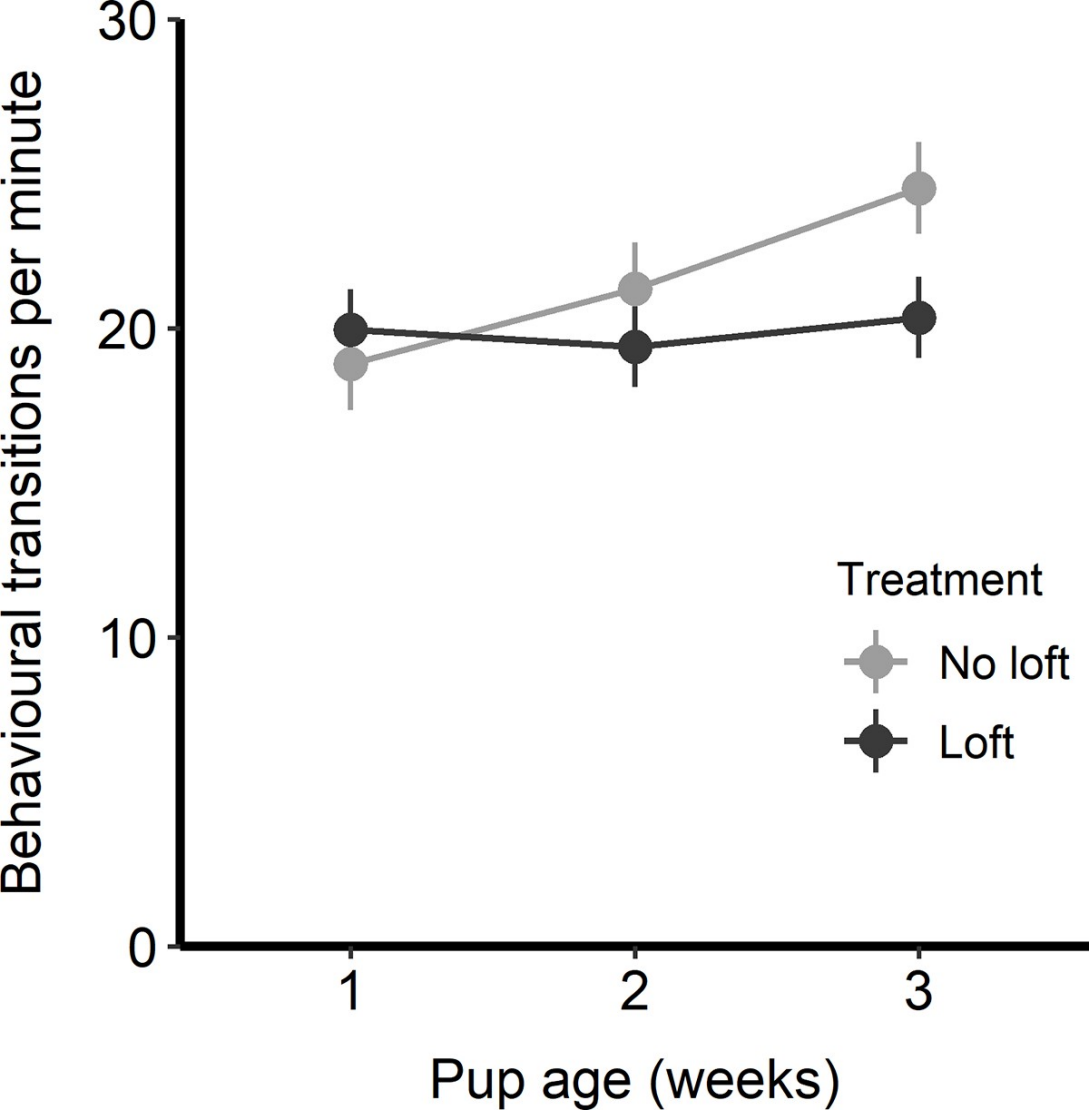
Behavioral transitions per minute during anticipatory behavior testing. Rats were assessed for this behavior twice per week and results were analyzed as weekly means. Results are shown separately by treatment. Plot depicts the LS means and standard errors from model output; n = 16 rats (9 in the loft treatment and 7 in the no loft treatment).

There was an overall effect of pup age (F_1,30_ = 5.05, p = 0.03) but we did note a tendency for an interaction between pup age and treatment (F_1,30_ = 3.81, p = 0.06), so the effect of pup age was analyzed separately by treatment. We found an effect of pup age on anticipatory behavior in the no loft group (F_1,13_ = 8.96, p = 0.01), while rats in the loft group showed no change in anticipatory behavior according to pup age (F_1,17_ = 0.05, p = 0.83). Anticipatory behavior was similar in the two treatments in week 1, but as pups became more mobile in weeks 2 and 3 the rats in the no loft treatment increased from 18.8 ± 1.0 behavioral transitions per minute in wk 1 to 24.5 ± 1.8 behavioral transitions per minute in week 3 (Fig 5). In contrast, rats in the loft treatment averaged approximately 20 behavioral transitions per minute with no effect of pup age.

### Elevated plus maze

**Fig 6:**
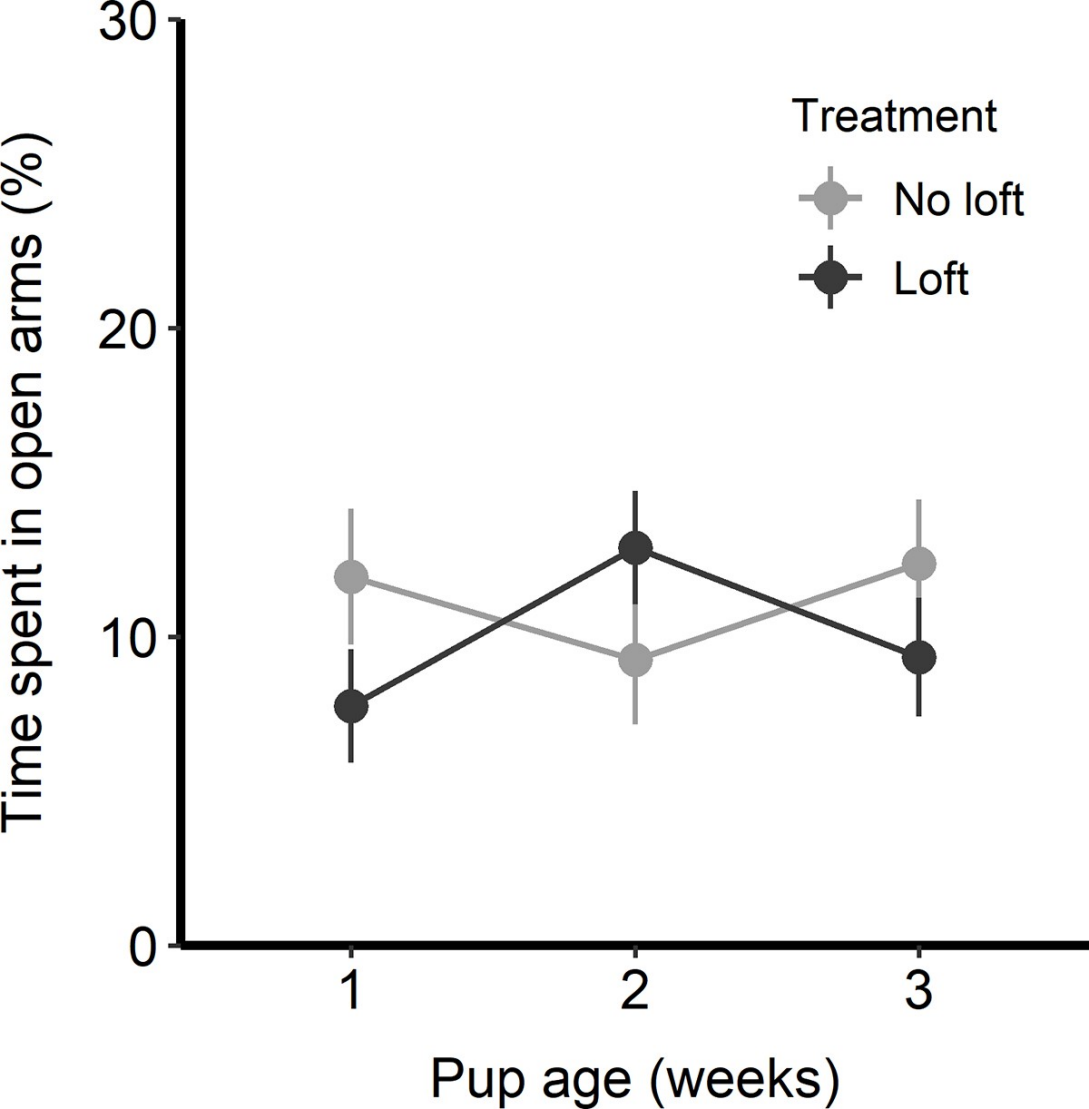
Mean time spent in the open arms during the elevated plus maze test. Rats were tested once per week over the pup rearing period. Results are shown separately by treatment with 9 rats in the loft treatment and 7 in the no loft treatment. Plot depicts the LS means and standard errors from the model output.

There was no effect of treatment (F_1,14_ = 0.36, p = 0.56), pup age (F_1,28_ = 0.42, p = 0.52), or interaction between treatment and pup age (F_1,28_ = 0.07, p = 0.79) on time spent in the open arms of the elevated plus maze. Rats varied between approximately 8% and 12% of time spent in the open arms of the EPM regardless of treatment (Fig 6).

## Discussion

Our results indicate that: 1) when provided the ability to spend time away from their pups, dams choose to do so, especially during the latter stages of lactation; 2) this ability to spend time in a loft did not decrease the amount of active nursing that pups received from the dam, but 3) it did decrease passive nursing (and thus also total nursing), especially during the second and third weeks of lactation. We saw no evidence of an effect of the loft treatment on EPM responses (suggesting no effect of treatment on anxiety), but did find an effect on anticipatory behavior, especially during the later stages of lactation (suggesting that the no-loft dams experienced a more negative affective state).

The pattern of loft use in our study follows that reported in previous work [1,10,11]: as pups aged, dams spent more time in the get-away area (Fig 2). Similarly, it has been shown that as pups age, rat dams in standard cages exhibit more press posture to reduce interactions with pups [13]. Access to the loft in the present study allowed the dam to reduce the amount of passive (and total) nursing provided to her litter. Our study is the first to examine the effects of a get-away loft in an existing standard cage, a refinement that has the potential to be widely applied. It has previously been shown that singly-housed rats in a multi-level laboratory cage preferentially sleep on the bottom level of the cage and perform active behavior in both the top and bottom levels; proportion of time spent in the upper level was not reported [27]. Additionally, prior studies reported only the patterns of contact or total amount of contact with pups, and did not assess the nature of these interactions. We have shown that a large proportion of nursing interactions in the treatment without the loft were initiated by pups (passive nursing) rather than actively provided by the dam; this result suggests that dams in standard laboratory housing experience more passive nursing than they would otherwise choose to endure. Some degree of weaning conflict is typical in mammals, with the young soliciting a higher level of parental investment than is ideal for the dam, a conflict which is expected to increase with age [28,29]. The get-away loft area in the current study provided dams with a level of control over their contact with pups. The ability to exert control over their environments, specifically choosing when and how to nurse, provides an aspect of agency that may be important for animal welfare. Špinka [3] argued that engagement in action-driven agency is associated with positive affective states, and opportunities for agency may contribute to more complete expression of species-typical behaviors.

A potential concern with providing dams the ability to avoid pups is that pups could receive less active maternal care and experience increased anxiety in the future, given that active maternal care has been linked with the development of stress responses in rat pups [30,31]. We did not find any difference between treatments in the level of active maternal care provided (Fig 3, panel A); dams with loft access provided active maternal care at a level similar to that of dams without the loft. Similarly, work on pigs has shown that sows with access to a get-away area were still responsive to offspring even though they were able to nurse less frequently [6].

The effect of the loft treatment may have been reduced by week 3, as over this period some pups had gained the ability to follow the dam up into the loft; it is likely that in the final days before weaning (i.e. days 19–21) the loft became less effective as a means for dams to spend time away from pups. This may also be the time when a get-away area is most valuable to the dams. Despite the potential decrease in treatment effectiveness, dams with loft access were still motivated to use it and spent roughly half their time in the loft during the third week, such that passive nursing was still lower in the loft treatment. Future work may choose to investigate designs that better allow dams to avoid older pups; for example Cramer et al. [12] separated compartments with a 30 cm wall that kept pups out until PND 30.

As predicted, rats without loft access showed an increase in anticipatory behavior as pups became more mobile, while rats in the loft were unaffected. Thus the inability to spend time away from pups may have been detrimental to the dam’s affective state, especially later in lactation. This result is consistent with the expected relationship between reward sensitivity and welfare described by van der Harst and Spruijt [19], with evidence of increased reward sensitivity when experiencing poor affect. This result is also consistent with the idea that animals with more control over their environment experience better affective states [3]. Alternatively, differences in anticipatory behavior could be related to motivation for food or preferences for a more complex environment. Rats that lacked a loft spent more time nursing, and as a result may have had increased energy requirements. Rats increase their food intake and frequency of meals during lactation, especially in the second and third weeks post partum [32]; although rats were fed *ad libitum*, motivation to eat more frequently, in combination with increased passive nursing, may have resulted in increased anticipatory behavior for the food reward. An alternate reward (such as access to an enriched cage without pups) may have helped to disentangle these effects. Another factor that may have played a role in anticipatory behavior is the general effect of decreased cage complexity for rats in the no loft treatment, given that rats were previously housed with lofts and had them removed during the study. A previous study found that removal of the loft in a double-decker rat cage negatively impacted the affective states of singly-housed rats [27].

Rearing (in combination with ‘rear-move’ behavior) was the most commonly displayed behavior during anticipatory behavior testing (S4 Table). Makowska and Weary [21] also found that rearing was the most common behavior shown by rats during anticipatory behavior testing. There is potential for future work focusing on the reliability of specific behaviors as indicators of anticipatory behavior in rats. It is not currently known whether specific behaviors are indicative of positive or negative emotions or if certain behaviors are more strongly linked to testing conditions; frequency of behavioral transitions is currently the most often used measure of anticipatory behavior, but has been criticized as non-specific (see [33] for a review).

Results of the EPM test showed no effect of treatment and thus did not support our prediction that rats with a loft would spend more time in the open arms. Rats were tested in the EPM at the end of each week, so week 3 tests were performed on PND 21. By this point pups had largely gained the ability to access the loft, such that the loft may have become less effective as a means of escaping pups. Another possible explanation for the lack of treatment effect in the EPM is habituation to the apparatus that may occur with repeated testing. Some measures of EPM behavior, such as overall movement and distance travelled, are more stable across trials; other measures more commonly used to measure anxiety, such as open arm avoidance, may be more susceptible to habituation with repeated exposures [26]. It is also possible that the inability to spend time away from pups did not affect dam anxiety or exploratory behavior.

### Limitations

Our sample contained dams of two different age groups; however, all rats were primiparous and there were no clear differences in behavior between older and younger dams. There was some variation in litter size and our study was not powered to test the interaction between litter size and treatment on dam behavior. Litter size may be an important co-variate and should be considered in future work. The diurnal cycle of maternal care is maintained independent of litter size [14], but prior studies have found effects of litter size on dam-pup interactions. Grota & Ader [1] found that time spent with the litter decreased more rapidly for females rearing litters of twelve compared to litters of four. Deviterne et al. [34] found that during the first 9 PND, rats with larger litters tended to spend more time with pups compared to rats rearing smaller litters. Gaskill and Pritchett-Corning [13] found that pups from larger litters weighed less at weaning than pups from smaller litters. In our study, most litters consisted of 12 pups, but 6 litters were smaller (3 to 10 pups).

Although we used a commercially available cage, the food hopper and hut were removed to help prevent pup access to the loft. A shelter is an important component of laboratory rodent housing, thus the setup in this study would not be ideal for widespread use. Including an effective get-away area for dams with an effective shelter for dams and pups may require creative alterations to the cage (e.g. creation of shelter insert to go underneath the loft area), or use of cages with more vertical space to increase the distance between the floor and the get-away area. It is possible that other commercially available cage models may better allow for a get-away area without removal of standard enrichment items (e.g. the Double Decker rat cage, Tecniplast, Italy).

Given that there are many potential impacts of the environment on lactating rats, it would be beneficial to research other welfare outcomes, including investigation of affective states using other approaches. Future work should examine press posture [13], as this may be another indicator of the suitability of a housing environment for rat dams. Anecdotally, this posture was observed by the experimenters during the light phase, but due to low frequency of the behavior during the period sampled, combined with difficulty scoring the behavior in videos due to the location of cage filters in the corners of the cages, this behavior was not included as an outcome measure. More frequent live scan sampling may have allowed for accurate monitoring of this behavior.

There are potential effects on offspring that were not assessed in the current study. For example, decreased opportunities for passive nursing may encourage pups to begin eating solid food at an earlier age. This has been demonstrated in pigs, where piglets raised by sows that spent time away show improved solid food consumption and weight gain after weaning [35]. Stress associated with abrupt weaning at 21 days of age may also be mitigated by gradual reduction of contact with the dam [28]. Pup weights at weaning are included as supplementary data (S4 Dataset), but we caution readers that the current study was not powered to detect treatment differences in this outcome.

## Conclusions

Our findings indicate that rats make use of get-away areas as a strategy to reduce passive nursing encounters and rats without access to a loft experience poorer affective states, particularly in the later stages of lactation.

## Supporting information

S1 Fig Cage set-up for each treatment, showing loft (A) and no-loft (B) treatments. Both cages contained aspen chip bedding, crinkle paper and paper towel as nesting materials, and food pellets on the cage floor.
(DOCX)

S1 Table Age, litter size, and treatment allocation for each individual rat. Results are shown separately by treatment with 9 rats in the loft treatment and 7 in the no loft treatment.
(DOCX)

S2 Table Dam weights (g). Dams were weighed at the end of each post-natal week. Data is missing for one rat in week 1.
(DOCX)

S3 Table Anticipatory behavior training progression. Each training trial was paired with a set delay time between the cue and provision of the reward. For the first ten trials, reward provision was immediately following the cue.
(DOCX)

S4 Table Mean ± SD frequency of each behavior scored during anticipatory behavior testing. n = 16 rats.
(DOCX)

S1 Dataset Data for time spent nursing (passive, active) and time spent in the loft. (CSV)

S2 Dataset Data for anticipatory behavior trials. (CSV)

S3 Dataset Data for elevated plus maze trials. (CSV)

S4 Dataset Data for pup weaning weights. (CSV)
