# Supplementary figures and images for: A break from the pups: The effects of loft access on the welfare of lactating laboratory rats
Source: PLoS One. 2021 Jun 8;16(6):e0253020. doi: 10.1371/journal.pone.0253020 (PMC8186774; doi:10.1371/journal.pone.0253020)

**S1 Figure. Cage set-up for loft (A) and no loft (B) conditions.**


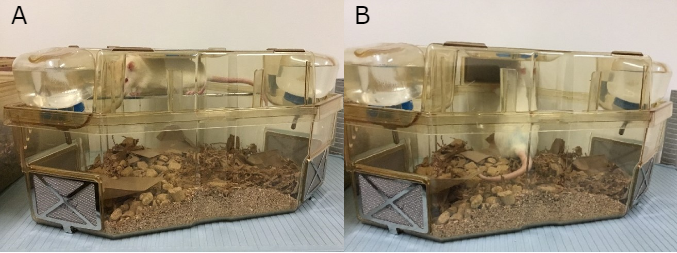

Supplement: S1 Fig — Cage set-up for each treatment, showing loft (A) and no-loft (B) treatments. Both cages contained aspen chip bedding, crinkle paper and paper towel as nesting materials, and food pellets on the cage floor. (DOCX) [file pone.0253020.s001.docx]
