# Supplementary material for: A break from the pups: The effects of loft access on the welfare of lactating laboratory rats
Source: PLoS One. 2021 Jun 8;16(6):e0253020. doi: 10.1371/journal.pone.0253020 (PMC8186774; doi:10.1371/journal.pone.0253020)
Supplement: S1 Table — Results are shown separately by treatment with 9 rats in the loft treatment and 7 in the no loft treatment. (DOCX) [file pone.0253020.s002.docx]

| Rat | Age | Treatment | Final litter size |
| --- | --- | --- | --- |
| 1 | 9 mo | L | 8* |
| 2 | 9 mo | NL | 12 |
| 7 | 9 mo | L | 12 |
| 4 | 9 mo | L | 12 |
| 9 | 9 mo | NL | 10 |
| 12 | 9 mo | NL | 12 |
| 20 | 4 mo | NL | 12 |
| 21 | 4 mo | L | 12 |
| 22 | 4 mo | L | 12 |
| 24 | 4 mo | NL | 12 |
| 25 | 4 mo | L | 6 |
| 26 | 4 mo | L | 9 |
| 28 | 4 mo | L | 12 |
| 29 | 4 mo | NL | 12 |
| 30 | 4 mo | NL | 3 |
| 31 | 4 mo | L | 7 |

**S1 Table. Age, litter size, and treatment allocation for each individual rat.** Results are shown separately by treatment with 9 rats in the loft treatment and 7 in the no loft treatment.

*denotes cross fostering occurred on PND 3 to increase litter size. L denotes loft, and NL denotes no loft treatment.
