# Supplementary material for: A break from the pups: The effects of loft access on the welfare of lactating laboratory rats
Source: PLoS One. 2021 Jun 8;16(6):e0253020. doi: 10.1371/journal.pone.0253020 (PMC8186774; doi:10.1371/journal.pone.0253020)
Supplement: S2 Table — Dams were weighed at the end of each post-natal week. Data is missing for one rat in week 1. (DOCX) [file pone.0253020.s003.docx]

**S2 Table. Dam weights (g).** Dams were weighed at the end of each post-natal week. Data is missing for one rat in week 1.

| Rat | Treatment | Week 1 | Week 2 | Week 3 |
| --- | --- | --- | --- | --- |
| 1 | L | 455 | 420 | 420 |
| 2 | NL | 410 | 425 | 400 |
| 4 | L | 460 | 445 | 416 |
| 7 | L | 445 | 485 | 455 |
| 9 | NL | 435 | 446 | 390 |
| 12 | NL | 440 | 413 | 395 |
| 20 | NL | 476 | 477 | 460 |
| 21 | L | 404 | 400 | 393 |
| 22 | L | 444 | 462 | 440 |
| 24 | NL | ---- | 424 | 395 |
| 25 | L | 398 | 408 | 409 |
| 26 | L | 390 | 397 | 380 |
| 28 | L | 484 | 490 | 480 |
| 29 | NL | 455 | 452 | 419 |
| 30 | NL | 409 | 410 | 396 |
| 31 | L | 449 | 449 | 406 |
