# Supplementary material for: A break from the pups: The effects of loft access on the welfare of lactating laboratory rats
Source: PLoS One. 2021 Jun 8;16(6):e0253020. doi: 10.1371/journal.pone.0253020 (PMC8186774; doi:10.1371/journal.pone.0253020)
Supplement: S3 Table — Each training trial was paired with a set delay time between the cue and provision of the reward. For the first ten trials, reward provision was immediately following the cue. (DOCX) [file pone.0253020.s004.docx]

**S3 Table.** **Anticipatory behavior training progression.** Each training trial was paired with a set delay time between the cue and provision of the reward. For the first ten trials, reward provision was immediately following the cue.

| Trial number | Delay between cue and reward (sec) |
| --- | --- |
| 1 | 0 |
| 2 | 0 |
| 3 | 0 |
| 4 | 0 |
| 5 | 0 |
| 6 | 0 |
| 7 | 0 |
| 8 | 0 |
| 9 | 0 |
| 10 | 0 |
| 11 | 5 |
| 12 | 10 |
| 13 | 15 |
| 14 | 20 |
| 15 | 25 |
| 16 | 30 |
| 17 | 35 |
| 18 | 40 |
| 19 | 45 |
| 20 | 50 |
| 21 | 60 |
| 22 | 90 |
| 23 | 120 |
| 24 | 150 |
| 25 | 180 |
