# Supplementary material for: A break from the pups: The effects of loft access on the welfare of lactating laboratory rats
Source: PLoS One. 2021 Jun 8;16(6):e0253020. doi: 10.1371/journal.pone.0253020 (PMC8186774; doi:10.1371/journal.pone.0253020)
Supplement: S4 Table — n = 16 rats. (DOCX) [file pone.0253020.s005.docx]

**S4 Table. Mean ± SD frequency of each behavior scored during anticipatory behavior testing.** n=16 rats.

| Behavior | Rate (mean ± SD) |
| --- | --- |
| Alert | 0.1±0.3 |
| Dig | 1.5±2.1 |
| Eat | 0.8±1.0 |
| Groom | 1.1±1.4 |
| Jump | 0.2±0.5 |
| Nesting material | 2.4±3.2 |
| Pups | 3.7±4.4 |
| Rear | 16.7±6.0 |
| Rear-move | 15.4±10.0 |
| Sit | 8.3±5.7 |
| Walk | 14.2±4.7 |
